# Gene expression in *Pseudomonas aeruginosa *swarming motility

**DOI:** 10.1186/1471-2164-11-587

**Published:** 2010-10-20

**Authors:** Julien Tremblay, Eric Déziel

**Affiliations:** 1 INRS-Institut Armand-Frappier, Laval (Québec), H7V 1B7, Canada

## Abstract

**Background:**

The bacterium *Pseudomonas aeruginosa *is capable of three types of motilities: swimming, twitching and swarming. The latter is characterized by a fast and coordinated group movement over a semi-solid surface resulting from intercellular interactions and morphological differentiation. A striking feature of swarming motility is the complex fractal-like patterns displayed by migrating bacteria while they move away from their inoculation point. This type of group behaviour is still poorly understood and its characterization provides important information on bacterial structured communities such as biofilms. Using GeneChip^®^ Affymetrix microarrays, we obtained the transcriptomic profiles of both bacterial populations located at the tip of migrating tendrils and swarm center of swarming colonies and compared these profiles to that of a bacterial control population grown on the same media but solidified to not allow swarming motility.

**Results:**

Microarray raw data were corrected for background noise with the RMA algorithm and quantile normalized. Differentially expressed genes between the three conditions were selected using a threshold of 1.5 log_2_-fold, which gave a total of 378 selected genes (6.3% of the predicted open reading frames of strain PA14). Major shifts in gene expression patterns are observed in each growth conditions, highlighting the presence of distinct bacterial subpopulations within a swarming colony (tendril tips vs. swarm center). Unexpectedly, microarrays expression data reveal that a minority of genes are up-regulated in tendril tip populations. Among them, we found energy metabolism, ribosomal protein and transport of small molecules related genes. On the other hand, many well-known virulence factors genes were globally repressed in tendril tip cells. Swarm center cells are distinct and appear to be under oxidative and copper stress responses.

**Conclusions:**

Results reported in this study show that, as opposed to swarm center cells, tendril tip populations of a swarming colony displays general down-regulation of genes associated with virulence and up-regulation of genes involved in energy metabolism. These results allow us to propose a model where tendril tip cells function as «scouts» whose main purpose is to rapidly spread on uncolonized surfaces while swarm center population are in a state allowing a permanent settlement of the colonized area (biofilm-like).

## Background

*Pseudomonas aeruginosa *is a ubiquitous Gram-negative rod-shaped bacterium responsible for many infections among immunocompromised hosts, burned patients and individuals suffering from cystic fibrosis. Besides well-known swimming and twitching motilities, this bacterium is capable of another type of migration called swarming. This complex type of motility is usually defined as a rapid and coordinated translocation of a bacterial population across a semi-solid surface (See additional file 1 (movie)) [1,2]. In addition to flagella, swarming of *P. aeruginosa *requires the release of two exoproducts, rhamnolipids (RLs) and 3-(3-hydroxyalkanoyloxy)alkanoic acids (HAAs), which act as wetting agents and chemotactic-like stimuli [3-6]. The best studied bacterial social behaviour is the formation of attached communities called biofilms. Besides playing a role in swarming motility, RLs and HAAs are also implicated in many aspects of biofilm development [7-9]. Interestingly, swarmer cells of a range of bacteria, including *P. aeruginosa *and *Salmonella typhimurium*, display enhanced resistance to a variety of antibiotics [10,11], a well-known feature of the biofilm way of life. A complex relationship exists between swarming motility and biofilm development [12-15].

A striking feature of *P. aeruginosa *colonies displaying swarming motility is the formation of complex dendritic, fractal-like patterns. Little is known about the gene regulation of the different bacterial subpopulation comprised in a swarming colony. Recently Overhage and coworkers [16] presented a microarray analysis in which a swarmer cell population harvested at the edge of a swarming colony migrating front was compared with broth cultured bacteria. Their data showed that swarm edge cells exhibited up-regulation of genes associated with virulence (*e.g*. Type III secretion system, extracellular proteases and iron transport) compared with cells cultured in broth.

Besides *P. aeruginosa*, the other bacterial species for which a transcriptomic study of the swarming state has been reported are *Salmonella typhimurium *[17], *Sinorhizobium meliloti *[18], and *Proteus mirabilis *[19]. In their experimental design, Wang *et al*., (2006) compared entire bacterial colonies grown on swarm medium to cells grown on hard surface not allowing swarming using broth culture for control [17]. They pointed out differentially expressed genes specific to the swarming motility of *Salmonella *and also determined that the expression of many genes associated with type III secretion, LPS synthesis and iron metabolism was surface-specific and not specifically associated with swarming. Very recently, Nogales *et al*., (2010) reported the transcriptome of *Sinorhizobium meliloti *grown on a semi-solid surface. One of their conclusions was that rhizobactin and iron metabolism genes play an important role in swarming motility of this bacterium [18]. The swarming transcriptome of *Proteus mirabilis *was also recently established [19]. Swarming motility in this bacterium displays concentric circles radiating from an inoculating point formed by successive swarming and consolidation phases and is quite different from what is observed in *P. aeruginosa*. The authors reported that flagellar genes were highly up-regulated in both swarming and consolidation cells compared to cells cultured in broth. Interestingly, comparison of these two phases revealed that only 9 genes were up-regulated during the swarm extension process.

Here we further dissect the whole-genome transcriptomic profile of *P. aeruginosa *swarming motility by comparing gene expression between different bacterial populations localized: 1) at the tip of migrating swarming tendrils, 2) at the center of a swarming colony, and 3) cultured in exactly the same growth conditions except for a harder surface not allowing this type of motility, as control.

## Methods

### Bacteria

This study was performed with *P. aeruginosa *strain PA14. Bacteria from frozen stocks were typically grown at 37°C in Tryptic Soy Broth (TSB) (Difco) in a rotary shaker. For swarming assays, overnight cultures were diluted in PBS to the desired OD_600_.

### Motility assays

Swarming motility assays were performed as previously described [20]. Medium M9DCAA [20 mM NH_4_Cl; 12 mM Na_2_HPO_4_; 22 mM KH_2_PO_4_; 8.6 mM NaCl; 1 mM MgSO_4_; 1 mM CaCl_2 _2 H_2_O; 11 mM dextrose; 0.5% casamino acids (Difco)] was solidified with 0.5% Bacto-agar (Difco) and dried for 60 min under laminar flow in two rows following the length axis of the laminar cabinet. Control non-swarming plates were identically prepared but instead dried for 240 min. All plates were inoculated at their center with 5 μL of cell suspension (OD_600 _= 3.0) and incubated at 30°C.

### Sample preparation for microarray hybridization

Actively migrating cells were harvested from the tip of migrating tendrils of swarming colonies 12 hrs post-inoculation by pipeting 8 μL of RNAlater (Qiagen) on the edge of a given tendril. Cells were resuspended by robustly pipeting back and forth while slightly inclining the Petri dish and were directly transferred into a 1.5 mL microtube kept on dry ice. An average of eight tendril migrating fronts per plate was harvested, with twenty plates for each replicate. Cells localized in the center of swarming colonies were harvested using a hole-cutter of 1 cm diameter and transferred into a clean Petri dish. Cells adhering to the agar plugs were then collected by vigorously pipeting back and forth 1 mL of RNAlater and transferred into a 1.5 mL microtube on dry ice. Ten swarm centers were sampled for each replicate. Non-swarming colonies were harvested by vigorously pipeting back and forth 1 mL of RNAlater directly on bacteria and were transferred into a 1.5 mL microtube kept on dry ice. Ten non-swarming colonies were use for each replicate.

RNA was extracted using the RiboPure™ kit (Ambion). All manipulations were performed according to the manufacturer's instruction. RNA purity was assessed by spectrophotometry (NanoDrop ND-1000). Samples showing ratios of A_260_/A_280 _and A_260_/_230 _superior to 2.0 were selected. RNA quality was then assessed with a Bioanalyzer 2100 (Agilent Technologies). Samples having a RIN of 8.9 and greater were kept.

To maximize cDNA synthesis yield, reverse transcription was performed twice with 6 μg of purified RNA for each replicate (total of 12 μg/replicate) using random hexamer primers (Invitrogen) and Superscript II reverse transcriptase (Invitrogen). GeneChip^®^ Eukaryotic Poly-A RNA Control Kit (Affymetrix) was integrated in each sample for quality control for the hybridization process. Thermocycler routine was performed according to Affymetrix's guidelines. RNA was eliminated with a HCl/NaOH 1N treatment and cDNA was purified with the MinElute PCR Purification Kit (Qiagen). Resulting cDNA was digested with DNaseI (Roche) to give fragments between 50 and 200 bases. Samples were tagged with Biotin with the GeneChip^®^ DNA Labeling Reagent (Affymetrix) according to the manufacturer's instructions. Quality control of biotin tagging was performed with a gel-shift assay using the ImmunoPure NeutrAvidin Protein (Pierce chemicals) and SybrGold (Invitrogen) for revelation.

### Microarray hybridization and data analysis

Hybridizations were performed at the Genome Québec Innovation Centre (McGill University, Montréal, Canada). Raw data were corrected for background using the RMA algorithm and quantile normalization[21]. Expression levels obtained from three replicates for each condition were compared using the FlexArray 1.3 software [22]. Only genes showing a *p*-value < 0.05 using the Empirical Bayes (Wright and Simon) algorithm were considered for data analysis. Since the RMA algorithm decreases the false positive rate and compresses the fold change, a 1.5-fold change cut-off value was used for determination of the differentially expressed genes [21]. Functional classification and over-representational analysis were performed using the PseudoCAP functional classes http://www.pseudomonas.com[23]. Expression data of all differentially expressed genes is available in additional file 2.

### qRT-PCR

Quantitative real-time PCR was performed using qScript™ One-Step SYBR Green kit (Quanta Bioscience) and a RotorGene 6000 thermocycler (Corbett). Primers were designed to give products between 80 and 150 bp. The *nadB *gene was used as a housekeeping control. Each qRT-PCR run was done in triplicate and for each reaction, the calculated threshold cycle (*C*t) was normalized to the *C*t of *nadB *amplified from the corresponding sample. The fold-change was calculated using the 2^-ΔΔCt ^method [24]. Sequences of primers used for qRT-PCR analysis are available in additional file 3.

## Results and Discussion

### Experimental design to identify differentially expressed genes in a *P. aeruginosa* swarming colony - significant transcriptional changes

Previous reports on swarming motility of *P. aeruginosa *showed that this social phenomenon relies on the expression of many genes [25-27]. These studies presented genes that are essential to swarming motility by screening transposon mutant libraries. This approach has provided precious insights but no information on the expression of genes involved in this multicellular behavior. The only published transcriptomic study on *P. aeruginosa *swarming motility was performed by Overhage and coworkers [16]. They reported that many genes associated with virulence were overexpressed at the swarming migration front compared to cells cultured in broth. However, because of the nature of their control condition (*i.e*. broth suspended cells), results reported in that study essentially gave information on transcriptional differences between surface and broth lifestyles.

Therefore, to further understand the complexity of *P. aeruginosa *swarming migration, microarrays gene expression profiles of bacterial populations localized at the tip of swarming tendrils and swarm centers were established using for control bacteria grown in the same culture conditions but on plates dried longer to prevent swarming motility [20]. Analysis of three independent experiments for each three conditions reveals a total of 378 differentially expressed genes (*p *< 0.05 by Empirical Bayes statistical test) using a threshold of +/-1.5 fold (log_2_) (table 1). These differently expressed genes are obtained by comparing each three conditions to one another: swarm center vs. non-swarming, tendril tip vs. non-swarming and tendril edge vs. swarm center. A selection of genes from our analysis is discussed below and presented in Tables 2, 3 and 4. A complete list of all differentially expressed genes is available (see additional file 2).

**Table 1:** Differentially expressed genes by pairwise comparison

	Tendril tip **vs**. non-swarming	Tendril tip **vs**. swarm center	Swarm center **vs**. non-swarming
**Genes up-regulated**	75	20	45
**Genes down-regulated**	232	121	43
**Total**	307	141	88

These three pairwise comparisons represent a total of 378 non-redundant genes.

**Table 2:** Selected genes up-regulated in tendril tip cells

Gene number	Gene name	Product name	**Tip vs. non-swarming fold change (log**_**2**_**)**
**Transcriptional regulators**			
PA0961^2^		probable cold-shock protein	1.5
PA5403^1^		probable transcriptional regulator	1.9
PA5550	*glmR*	GlmR transcriptional regulator	1.6
			
**Energy metabolism**			
PA1552	*ccoP1*	probable cytochrome c	1.9
PA1553	*ccoO1*	probable cytochrome c oxidase subunit	2.1
PA1554	*ccoN1*	probable cytochrome oxidase subunit (cbb3-type)	1.7
PA4133^2^		cytochrome c oxidase subunit (cbb3-type)	2.9
PA4429	*petC*	probable cytochrome c1 precursor	2.0
PA4430	*petB*	probable cytochrome b	2.0
PA4431	*petA*	probable iron-sulfur protein	1.5
PA5553	*atpC*	ATP synthase epsilon chain	1.5
PA5554	*atpD*	ATP synthase beta chain	1.9
PA5555	*atpG*	ATP synthase gamma chain	1.7
PA5556*	*atpA*	ATP synthase alpha chain	1.4
PA5557*	*atpH*	ATP synthase delta chain	1.3
PA5558*	*atpF*	ATP synthase B chain	1.3
PA5559*	*atpE*	ATP synthase C chain	1.3
PA5560	*atpB*	ATP synthase A chain	1.8
PA5561*	*atpI*	ATP synthase protein I	1.3
			
**Protein secretion/export apparatus**			
PA3821	*secD*	secretion protein SecD	1.6
PA3820	*secF*	secretion protein SecF	1.6
PA5568	*yidC*	Preprotein translocase subunit YidC	1.7
			
**Transport of small molecules**			
PA3531^1^	*bfrB*	bacterioferritin	2.7
PA4616^1^		probable c4-dicarboxylate-binding protein	2.2
PA3187	*gltK*	probable ATP-binding component of ABC transporter	1.5
PA3188	*gltG*	probable permease of ABC sugar transporter	2.0
PA4628	*lysP*	lysine-specific permease	1.7
PA5479	*gltP*	proton-glutamate symporter	1.6
PA0782^3^	*putA*	proline dehydrogenase	1.8
PA0783^1^	*putP*	sodium/proline symporter PutP	1.6
PA4770^1^	*lldP*	L-lactate permease	1.5
			
**Translation, post-translational modification, degradation**			
PA0579	*rpsU*	30S ribosomal protein S21	1.5
PA2619	*infA*	initiation factor	1.8
PA2851	*efp*	translation elongation factor P	1.8
PA3655^1^	*tsf*	elongation factor Ts	1.7
PA3742	*rplS*	50S ribosomal protein L19	1.8
PA4255	*rpmC*	50S ribosomal protein L29	1.7
PA4432^1^	*rpsI*	30S ribosomal protein S9	1.8
PA4567	*rpmA*	50S ribosomal protein L27	1.5
PA4672		peptidyl-tRNA hydrolase	1.7
PA5049	*rpmE*	50S ribosomal protein L31	1.6

^1^: Genes up-regulated in tendril tip vs. NS and up-regulated in tendril tip vs. center.

^2^: Genes up-regulated in tendril tip vs. NS and up-regulated in swarm center vs. NS.

^3^: *putA *was significantly up-regulated in tip cells vs. swarm center, but not tip cells vs. NS.

*: Genes up-regulated in tendril tip vs. NS only and expressed more than 1.3 log_2_-fold). Genes kept in our analysis since they are part of an operon or in certain cases, a pertinent cluster.

**Table 3:** Selected genes down-regulated in tendril tip cells

Gene number	Gene name	Product name	**Tip vs. non-swarming fold change (log**_**2**_**)**
**Adaptation, Protection**			
PA2147^1^	*katE*	catalase HPII	-3.2
			
**Chemotaxis**			
PA1930^1^	*mcpS*	probable chemotaxis transducer	-2.8
PA2788^2^		probable chemotaxis transducer	-2.3
PA4915		probable chemotaxis transducer	-1.6
			
**Biosynthesis of cofactors, prosthetic groups and carriers**			
PA1985	*pqqA*	pyrroloquinoline quinone biosynthesis protein A	-2.2
PA1986	*pqqB*	pyrroloquinoline quinone biosynthesis protein B	-1.9
PA1987	*pqqC*	pyrroloquinoline quinone biosynthesis protein C	-1.8
PA1988^2^	*pqqD*	pyrroloquinoline quinone biosynthesis protein D	-2.2
PA1989	*pqqE*	pyrroloquinoline quinone biosynthesis protein E	-1.6
			
**Energy metabolism**			
PA0105^2^	*coxB*	cytochrome c oxidase, subunit II	-2.9
PA0106^2^	*coxA*	cytochrome c oxidase, subunit I	-2.7
PA0107^2^		conserved hypothetical protein	-2.2
PA0108^2^	*coIII*	cytochrome c oxidase, subunit III	-1.9
PA1175^2^	*napD*	NapD protein of periplasmic nitrate reductase	-2.2
PA1177^2^	*napE*	periplasmic nitrate reductase protein NapE	-2.3
PA1931		probable ferredoxin	-2.2
PA2153^2^	*glgB*	1,4-alpha-glucan branching enzyme	-3.6
PA2165^2^		probable glycogen synthase	-2.9
PA2290^2^	*gcd*	glucose dehydrogenase	-1.6
PA3416		probable pyruvate dehydrogenase E1 component, beta chain	-1.9
PA3417		probable pyruvate dehydrogenase E1 component, alpha subunit	-1.6
PA5427	*adhA*	alcohol dehydrogenase	-2.2
			
**Secreted Factors (toxins, enzymes, alginate)**			
PA2570^2^	*lecA*	LecA	-4.5
PA1148	*toxA*	exotoxin A precursor	-1.5
PA1245^2^	*aprX*	Hypothetical protein	-2.5
PA1246^2^	*aprD*	alkaline protease secretion protein AprD	-3.0
PA1247	*aprE*	alkaline protease secretion protein AprE	-1.6
PA1249	aprA*	alkaline metalloproteinase precursor	-1.4
PA1250^2^	*aprI*	alkaline proteinase inhibitor AprI	-1.9
PA1871^2^	*lasA*	LasA protease precursor	-3.2
PA2939^3^	*pepB*	Aminopeptidase	-1.5
PA1130	*rhlC*	rhamnosyltransferase 2	-1.5
PA1131		probable major facilitator superfamily (MFS) transporter	-1.6
PA3478	*rhlB*	rhamnosyltransferase 1	-2.3
PA3479^2^	*rhlA*	HAA synthase	-2.0
PA2255^3^	*pvcB*	paerucumarin biosynthesis protein PvcB	-1.7
PA2402	*pvdI*	non-ribosomal peptide synthase PvdI	-1.5
PA2406^3^		hypothetical protein	-1.9
PA2408^3^		probable ATP-binding component of ABC transporter	-1.7
PA2411^2^		probable thioesterase	-1.7
PA2412^2^		hypothetical protein	-1.6
PA2413^2^	*pvdH*	L-2,4-diaminobutyrate:2-ketoglutarate 4-aminotransferase, PvdH	-2.0
PA2424^2^	*pvdL*	Predicted non-ribosomal peptide synthetase PvdL	-2.3
PA2425	*pvdG*	Thioesterase PvdG	-1.8
PA4222	*pchI*	probable ATP-binding component of ABC transporter	-2.0
PA4223^2^	*pchH*	probable ATP-binding component of ABC transporter	-2.0
PA4224^2^	*pchG*	pyochelin biosynthesis protein PchG	-2.3
PA4225^2^	*pchF*	pyochelin synthetase	-2.4
PA4226^2^	*pchE*	dihydroaeruginoic acid synthetase	-2.2
PA4228	*pchD*	pyochelin biosynthesis protein PchD	-1.7
PA4229^2^	*pchC*	pyochelin biosynthetic protein PchC	-1.8
PA4230	*pchB*	salicylate biosynthesis protein PchB	-1.8
PA4231	*pchA*	salicylate biosynthesis isochorismate synthase	-1.8
			
**Transcription factors**			
PA2259	*ptxS*	transcriptional regulator PtxS	-2.0
PA0471	*fiuR*	Anti-sigma factor for FiuI	-1.9
PA0472	*fiuI*	sigma-70 factor, ECF subfamily	-1.5
PA1300		sigma-70 factor, ECF subfamily	-1.9
PA2895		Anti-sigma factor for PA2896	-1.9
PA2896		sigma-70 factor, ECF subfamily	-1.8
PA1912		sigma-70 factor, ECF subfamily	-1.8
PA2312		probable transcriptional regulator	-1.7
PA5116		probable transcriptional regulator	-1.6
			
**Two-component regulatory systems**			
PA3346		probable two-component response regulator	-1.6
PA1243		probable sensor/response regulator hybrid	-2.2
PA2177		probable sensor/response regulator hybrid	-1.8

^1^: Genes down-regulated in tendril tip vs. NS and down-regulated in swarm center vs. NS.

^2^: Genes down-regulated in tendril tip vs. NS and down-regulated in tips vs. swarm center.

^3^: Genes down-regulated in tendril tip vs. swarm center only.

*:*aprA *was kept since it is part of a pertinent gene cluster found in our analysis.

**Table 4:** Selected genes up-regulated in swarm center

Gene number	Gene name	Product name	**Swarm center vs. non-swarming fold change (log**_**2**_**)**
**Adaptation, Protection**			
PA0140^1^	*ahpF*	alkyl hydroperoxide reductase subunit F	4.5
PA0848^1^		probable alkyl hydroperoxide reductase	5.4
PA0849^1^	*trxB2*	thioredoxin reductase 2	3.7
PA3287^1^		conserved hypothetical protein	4.7
PA4236^1^	*katA*	catalase	3.4
PA4613^1^	*katB*	catalase	4.4
			
**Energy metabolism**			
PA4133^2^		cytochrome c oxidase subunit (cbb3-type)	1.6
			
**Hypothetical, unclassified, unknown**			
PA3237^1^		hypothetical protein	5.4
PA3287^1^		hypothetical protein	4.7
PA3519^1^		hypothetical protein	3.6
PA3520^3^		hypothetical protein	1.8
			
**Nucleotide biosynthesis and metabolism**			
PA5541^1^	*pyrQ*	dihydroorotase	2.5
			
**Transcriptional regulators**			
PA4878^1^		probable transcriptional regulator	3.7
			
**Transport of small molecules**			
PA2322	*gntT*	gluconate permease	2.0
PA3187^2^	*gltK*	ATP-binding component of ABC transporter	2.6
PA3188^2^	*gltG*	permease of ABC sugar transporter	3.1
PA3189	*gltF*	permease of ABC sugar transporter	2.5
PA3523^1^	*mexP*	probable Resistance-Nodulation-Cell Division (RND) efflux membrane fusion protein precursor	4.2
PA3920^1^	*cueA*	copper homeostasis P-type ATPase	2.6
PA5082		probable binding protein component of ABC transporter	1.5

^1^: Genes up-regulated in swarm center vs. NS and up-regulated in swarm center vs. tendril tip.

^2^: Genes up-regulated in swarm center vs. NS and up-regulated in tendril tip vs. NS (Table 1).

^3^: Gene up-regulated in swarm center vs. tendril tip only.

Pairwise comparisons of differentially expressed genes show that a majority of them were differentially expressed in the tendril tip vs. non-swarming category followed by tendril tip vs. swarm center then swarm center vs. non-swarming, respectively (Table 1). Interestingly, many more genes were repressed in tendril tip bacteria (232) compared to the up-regulated ones (75) in the tendril tip vs. non-swarming category, while 121 genes were down-regulated and 20 up-regulated in tendril tip compared to swarm center. The other category (swarm center vs. non-swarming) showed a more even distribution of up-regulated (45) and down-regulated (43) genes. To further expose their complexity, the expression data have been scattered in a Venn diagram (additional file 4). Genes ID and fold-change (log_2_) values can be found in additional file 2 (excel file) under the *summary *tab.

The most striking aspect of the expression data overview is that an important majority of differently expressed genes are down-regulated in the tendril tip populations compared to the non-swarming control and to the swarm center. Furthermore, there are far fewer genes differently expressed between the swarm center and non-swarming control that there are between tendril tip and non-swarming control. This suggests that swarm center cells are metabolically closer to non-swarming condition cells than to tendril tip cells. Interestingly, the few differently expressed genes present in the swarm center vs. non-swarming category in general present a higher fold-change value that those observed in the tendril tip vs. non-swarming category.

**Figure 1:**
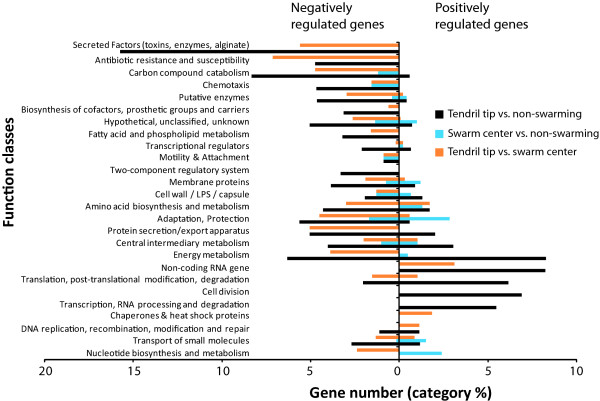
**Global gene expression pattern with a change in expression level greater than 1.5 log_2_-fold in the three tested conditions**. Overrepresentation analysis in functional class percentage for each PseudoCAP function classes according to up- and down-regulated genes showing the differential regulation of all gene classes in tendril tip vs. non-swarming, swarm center vs. non-swarming and tendril tip vs. swarm center.

Figure 1 presents an over-representation analysis of gene expression data based on their PseudoCAP function classes [23] in function of class category %. We can see that many genes belonging to the Secreted factors (toxins, enzymes, alginate) and Carbon compound catabolism categories are down-regulated in tendril tip cells. On the other hand, swarming tendril tip populations up-regulate genes in the Translational, post-translational modification, degradation, Non-coding RNA gene, Cell division and Transcription, RNA processing and degradation categories. The Energy metabolism category is also disregulated in swarm tip cells. Also noteworthy is that the tendril tip vs. non-swarming category shows a majority of genes down-regulated in membrane proteins, transport of small molecules, two-component regulatory systems, biosynthesis of cofactors, prosthetic groups and carriers and transcriptional regulators. It is to be noted that no genes coding for products of the flagellar apparatus are seen in our analysis. This agrees with our microscopic observations of PA14 tendril tip and swarm center cells, which always show single flagella-equipped bacteria (additional file 5).

### Validation of microarray results by qRT-PCR

**Figure 2:**
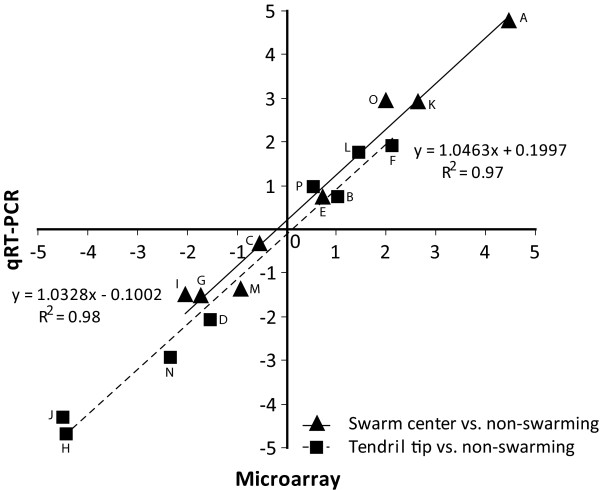
**Microarray results validation by qRT-PCR**. Mean log_2 _ratios of the qRT-PCR experiments are plotted against the mean log_2 _ratios of the microarray experiments. Numbers on the graph refer to genes listed in Table 5.

Validation of microarray data was performed using qRT-PCR. Eight genes in tendril tip vs. non-swarming (1 up-, 4 down-regulated and 3 non-differentially expressed genes) and swarm center vs. non-swarming (3 up-, 2 down-regulated and 3 non-differentially expressed genes) were selected for this comparative analysis (Table 5). Expression data of microarray and qRT-PCR are plotted in figure 2 and demonstrate an excellent concordance between the two datasets. Pearson correlation values scored 0.97 and 0.98 for center vs. non-swarming and vs. tendril tip, respectively.

**Table 5:** Genes used for microarray validation with qRT-PCR

Gene number	Gene name	Product name	**MA fold change (log**_**2**_**)**		**qRT-PCR fold change (log**_**2**_**)**	
			Center **vs**. non-swarming	Tip **vs**. non-swarming	Center **vs**. non-swarming	Tip **vs**. non-swarming
PA0140	*aphF*	alkyl hydroperoxide reductase subunit F	^A^4.5	^B^1.0	^A^4.7	^B^0.7
PA1130	*rhlC*	rhamnosyltransferase 2	^C^-0.5	^D^-1.5	^C^-0.4	^D^-2.1
PA1553	*ccoO1*	probable cytochrome c oxidase subunit	^E^0.7	^F^2.1	^E^0.7	^F^1.9
PA2158		probable alcohol dehydrogenase (Zn-dependent)	^G^-1.7	^H^-4.4	^G^-1.5	^H^-4.7
PA2570	*lecA*	LecA	^I^-2.0	^J^-4.5	^I^-1.5	^J^-4.3
PA3187		probable ATP-binding component of ABC transporter	^K^2.6	^L^1.5	^K^2.9	^L^1.8
PA3478	*rhlB*	rhamnosyltransferase chain B	^M^-0.9	^N^-2.3	^M^-1.4	^N^-2.9
PA5540		Hypothetical protein	^O^; 2.0	^P^0.5	^O^2.9	^P^1.0

Letters for each value refer to the corresponding points in figure 2.

### Genes positively regulated in tendril tip cells

Among the genes up-regulated in tendril tip bacterial population are found a high number of genes involved in the energy metabolism functional class. These genes' products include many cytochromes (PA1552-PA4429-PA4430) and cytochrome oxidase subunits (PA1553-PA1554-PA4133), which are involved in the production of ATP via the respiratory electron transport chain. We also found the *atpIBEFHAGFC *cluster coding for the only ATP synthase complex of *P. aeruginosa*. This suggests that fast moving swarmer cells in tendril tips require more energy than non-swarming and swarm center cells.

Bacterial heme-copper oxidases, such as cytochrome *c *oxidases, are key components of cellular energy transduction systems and contribute to the establishment of the electrochemical gradient subsequently used for ATP production by the ATP synthases [28]. At the tip of swarming tendrils, PA4429-31 is overexpressed (Table 2). This operon shows high similarity to *petABC *(also known as *fbcFBC*) encoding for polypeptides of the cytochrome *bc1 *complex (ubihydroquinone: cytochrome *c *oxidoreductase) [29]. A third group of cytochromes having a very high affinity for O_2 _has been described in recent years among proteobacteria, the cytochrome *cbb_3 _*oxidase [30]. *P. aeruginosa *contains two *ccoNOQP *operons (*ccoNOQP-1 *and *ccoNOQP-2*) coding for cytochrome *cbb*_3 _oxidases (PA1552-PA1554 and PA1555-PA1557, respectively). Intriguingly, *ccoNOQP-1*, but not *ccoNOQP-2*, is up-regulated in tendril tip cells (Table 2). Most studies on several bacterial *cbb_3 _*oxidases have indicated that these enzymes are primarily expressed under oxygen limitation and are critical for respiration in microaerobic conditions. However, the *P. aeruginosa *homologues show different expression patterns depending on oxygen availability [31]: the first operon (*ccoNOQP1*) displays higher expression under high oxygen availability while the second one is up-regulated under oxygen-limiting condition [31]. Actually, recent results indicate that Cbb3-1 plays a primary role in aerobic growth irrespective of oxygen concentration; Kawakami *et al*. (2009) have observed a phase-dependent regulation of *ccoNOQP-1 *expression, with much higher transcription levels in exponential phase compared to stationary phase growth [32].

In the end, our data indicates that two cytochrome *c *oxidases are specifically induced in tendrils tip cells; they are not differentially regulated in the swarm center and non-swarming conditions. Since the ATP synthase gene cluster is also up-regulated at the tip (Table 2), we conclude that swarming tip cells are highly metabolically active; their elevated energy requirements, presumably mostly for motility purposes, involve elevated O_2 _consumption.

We also obtained an up-regulation of many ribosomal proteins in tendril tip cells. Ribosomal proteins assist in the assembly and increase the stability of rRNA, without requiring ATP for their action [33]. Synthesis of ribosomal proteins and rRNA is tightly regulated and coordinated so they are never in excess [34]. This, along with the up-regulation of many tRNA genes in tendril tips (see additional file 2), suggests that tip cells display a high protein synthesis rate. Swarming cells of *S. meliloti *were also reported to display an up-regulation of many ribosomal proteins [18].

A number of tendril tip overexpressed genes are involved in the transport of small molecules (Table 2). Among them, *bfrB *was up-regulated 2.74 log_2_-fold. This gene codes for a bacterioferritin, a protein involved in the controlled storage and release of iron [35] that acts as a buffer against iron overload and deficiency [36]. An investigation of the global transcriptional response of iron-starved cultures of *P. aeruginosa *to iron exposure showed that mRNA levels of *bfrB *increased significantly when iron was made available, whereas *bfrA *mRNA levels remained unchanged [37]. Since tendril migrating front cells are colonizing still unpopulated (*i.e*. iron rich) area, *bfrB *up-regulation is in agreement with previous findings that BfrB is induced by iron-replete conditions while BfrA is constitutively expressed [37,38].

Both the proline and the glutamate symporters (*putP *and *gltP*) are induced at the swarming tip. In bacteria, glutamate serves as the general amino group donor for amino acid and nucleotide biosynthesis, and may also act as a source of carbon and nitrogen under nitrogen-limiting conditions. In many bacteria, proline is needed as an osmoprotectant in growth environments with high osmotic stress [39]. It is also catabolically converted into glutamate by the product of *putA *(encoding for a proline dehydrogenase) [40] which was also up-regulated in tip cells. Activation of the lysine permease and genes coding for the GltF-GltG-GltK high-affinity glucose transporter [41] further support a general up-regulation of specific uptake mechanisms in metabolically very active cells located at the tip of swarming tendrils.

Two secretion-related genes, *secD *and *secF*, were up-regulated in tendril tips. In *E. coli*, SecD/SecF are required for the proton-motive force dependant translocation of proteins [42]. Why only *secD *and *secF *and not other *sec *genes are differentially expressed is elusive at the moment. In *E. coli*, YidC associates with SecD and SecF in a preprotein translocase [43]. YidC depletion dissipated proton motive force, notably caused by defects in membrane assembly of both cytochromes and ATPases [44]. The up-regulation of *secD secF*, *and yidC *might thus be related to, and a consequence of, the induced cytochrome and ATPase components in swarm tip cells.

### Genes negatively regulated in tendril tip cells

Interestingly, the global picture of expression data shows that most disregulated genes found in our study are actually down-regulated in cells located at tendril tips compared to the non-swarming control (Figure 1A). Among them, we found the *coxBAC-colI *operon encoding for the *aa_3 _*cytochrome *c *oxidase [32]. Expression of this operon is up-regulated at the stationary phase, especially by nutrient-limiting conditions: carbon, nitrogen and iron starvation all induce transcription from the *cox *promoter [32]. Not surprisingly, the stationary-phase sigma factor RpoS is a positive regulator of the *cox *genes [45]. It is thus perfectly coherent with the model that swarming tip bacteria are highly active cells growing under nutrient-replete conditions.

The entire *pqq *operon (*pqqDABCE*) coding for pyrroloquinoline quinone (PQQ) was down-regulated in tendril tips. PQQ is one of several quinone derivatives functioning as essential cofactors for a class of enzymes known as quinoproteins [46,47]. For instance, it is the co-factor of the glucose dehydrogenase which catalyses the conversion of glucose to gluconate [47], the first step in the oxidative pathway of glucose utilisation. Interestingly, the *gcd *(PA2290) gene encoding for the membrane-bound glucose dehydrogenase is also down-regulated in tendril tips (Table 3). This would mean that swarming tip cells prefer to uptake and use glucose directly through the phosphorylative pathway to sustain the Entner-Doudoroff pathway. The phosphorylative pathway is thought to be preferred when oxygen is limiting [48]. Explanation to why *pqq *and *gcd *are down-regulated in tendril tip bacteria remains speculative at the moment: maybe the high respiration rate and high energy consumption at the tip of tendrils can somehow be reflected in reduced O_2 _availability and switching to the phosphorylative pathway of glucose utilisation.

Genes with chemotaxis-related functions were also found to be down-regulated. PA1930 codes for the soluble chemotaxis transducer McpS that was shown to be localized at cell poles [49]. It was also reported that the N terminus of McpS carries two putative PAS domains. These domains are present in numerous MCPs involved in light, oxygen and redox sensing [49]. The role of PA2788 and PA4915, two probable chemotaxis transducers is still unknown.

PA2165, coding for a probable glycogen synthase and *glgB*, encoding a glucan branching enzyme were also both down-regulated in tendril migrating front. These enzymes are involved in building glucose polymeric stocks (*i.e*. glycogen). This suggests that tendril tip bacteria are not building energy reserves but are instead consuming available carbon sources immediately.

An important observation is that many genes associated with virulence, especially secreted factors, were down-regulated in tendril tips and swarm center vs. non-swarming conditions. The extensive damage caused by *P. aeruginosa *during infections is due to the production of several cell-associated and extracellular virulence factors [50]. Interestingly, the previously published genome-wide transcriptomic study reported that swarming cells of *P. aeruginosa *display enhanced expression of many virulence determinants [16]. For instance, these authors found that genes associated with pyoverdin, pyochelin and phenazine biosynthesis were up-regulated in migrating swarm front compared to broth cultured cells [16]. In contrast, our transcriptomic data indicates that under swarming motility (tendril tip cells) there is a global shutdown of this category of genes.

We did not expect *rhlAB *and *rhlC *to be down-regulated in swarm tendril tips since rhamnolipid production is a key factor in *P. aeruginosa *swarming motility. However, close examination of the expression data actually reveals that *rhlAB *is more highly expressed in cells from the swarm center than from the tendril tips, suggesting that rhamnolipids are primarily produced from cells at the center of a swarming colony.

Pyochelin (*pchI*, *pchH*, *pchG*, *pchF*, *pchE*, *pchD*, *pchC*, *pchB*, *pchA*) and pyoverdin (*pvdI*, *pvdH, pvdLG*, PA2403-2410 operon, PA2411, PA2412) synthesis genes were also down-regulated in tendril tip cells. Pyochelin and pyoverdin are the two major siderophores produced by *P. aeruginosa *to acquire iron [51-53]. They chelate iron in the extracellular medium and transport it into the cells via specific outer membrane transporters, FptA for pyochelin [54] and FpvA for pyoverdin [55]. Besides iron, pyochelin also has affinity for other metals such as Co^2+^, Ga^3+^, and Ni^2+ ^[56]. As it is the case with *rhlAB *and *rhlC*, the *pch *and *pvd *genes were more highly expressed in swarm center compared to tendril tips, possibly because the cellular density in the swarm center is higher and that iron availability is therefore depleted and restricted. Indeed swarming tendrils are moving towards uncolonized areas that are still rich in iron (and other metals and nutrients), thus possibly explaining the down-regulation of siderophore synthesis genes. This is consistent with the above noted up-regulation of the bacterioferritin coding gene *bfrB *at the tip of tendrils. In addition, we previously demonstrated that rhamnolipids/HAAs diffuse rapidly on a surface [6,20]. These surfactant molecules are known to be potent antimicrobials and it is possible that one function of swarm center cells is to produce a quickly diffusing rhamnolipid-rich area protecting the tendril tip bacteria migrating in a hostile environment.

The expression of a number of protease-encoding genes is reduced at the tip of tendrils. Genes *lasA *and PA2939, which respectively encodes for the LasA protease, a 20-kDa staphylolytic enzyme [57], and a secreted aminopeptidase [58] were down-regulated in tendril tip cells. Genes belonging to the alkaline protease synthesis and secretory cluster (*aprXaprDaprE, aprA, aprII*) were also down-regulated in tendril migrating front cells. The expression of these genes is up-regulated under iron-limiting conditions [59].

The two genes *ptxS *and *toxA *were also down-regulated in tendrils. PtxS is a transcriptional regulator that controls the expression of *toxA *[60,61], whose product exotoxin A, like many other bacterial virulence factors, is negatively regulated by iron availability [59,62-64]. Again, this is consistent with tendril tip cells migrating over an uncolonized area where iron resources have not been yet depleted.

The gene coding for the galactophilic lectin LecA was down-regulated at the tendril tips. The product of this gene is an adhesin which has the ability to bind cells together in a biofilm [65]. The individual nature of fast moving swarming cells makes it logical for *lecA *to be down-regulated under these conditions, to allow cells to freely migrate.

Apart from PA5403 and PA5550 (*glmR*/*glpR*) (Table 2), nearly all genes belonging to the transcription factors class were down-regulated in tendril tip populations (Table 3), including a number of ECF sigma factors. GlmR is a regulatory gene involved in amino sugar metabolism. Its inactivation abolished swarming, swimming and twitching motilities of *P. aeruginosa *strain PA01 [66], but not of strain PA14 using our swarming conditions (data not shown). It was also reported that *glmR *(*glpR*) shows increased expression during twitching-mediated chemotaxis towards phosphatidylethanolamine [67]. The ECF sigma factor-putative anti-sigma factor couple PA2895-96 was identified to have a role in the secretion of exoproteases [25], and might be under iron regulation [68]. ECF sigma factors encoded by PA1300, PA1912 and *fiuI*, the latter along with its related anti-sigma factor coding *fiuR *[69], are up-regulated by iron starvation [59,70]. Again, the down-regulation of these genes in tip cells indicates they are under an iron-replete environment.

### Genes up-regulated in the swarm center

A substantial number of genes were highly up-regulated in swarm center (most with log_2_-fold > 3) compared to the non-swarming control. Among the three conditions tested in this study, the comparison between swarm center and non-swarming displays the least expression differences, as shown by the low category % of differentially expressed genes (Figure. 1; blue bars). This suggests that these two conditions display a more similar biological status (*i.e*. as opposed to tendril tip vs. non-swarming and tendril tip vs. swarm center). However, as reported in table 4, the few swarm center genes considered in our analysis were on average much more highly differentially expressed (*i.e*. average log_2_-fold change > 3.5).

*P. aeruginosa *codes for three different catalases. KatA is the major catalase which is highly expressed in all phases of growth [71]. KatB is detectable when induced by peroxide or paraquat [72]. The KatE (aka KatC) catalase is high-temperature inducible [73]. However its function is unclear as it does not contribute significantly to protection against oxidative stress and high osmolarity, or to virulence, under standard laboratory conditions [74][375]. Both *katA *and *katB *were strongly induced in the swarm center vs. non-swarming conditions (Table 4). In contrast, the catalase-encoding gene *katE *was down-regulated in swarming tendril tip cells (Table 3). Furthermore, alkyl hydroperoxide reductase AhpF, the thioredoxin reductase 2 (*trxB2*) and PA3237 were also up-regulated in swarm center. The latter codes for a predicted protein of about 8 kDa with an export signal. Along with *katA *and *katB*, these three genes are among the more strongly induced genes by H_2_O_2 _exposure [75], clearly suggesting the occurrence of such stress in the center of a swarming colony. Finally, the expression of PA3287 was also reported to be induced by H_2_O_2 _[76]. This gene shares high similarity with ankyrin. Interestingly, AnkB, an ankyrin-like protein, is needed for optimal KatB activity [77].

Many genes associated with the copper stress response (*mexP*, cueA, PA3519-20) [78] were highly up-regulated in the swarm center population. These genes are part of a small subset of genes reported to be directly regulated by CueR, a transcriptional regulator central to copper resistance in *P. aeruginosa *[79]. While copper is an important element in cellular metabolism, Cu^2+ ^was shown to accumulate in the EPS matrix of biofilms and to be particularly toxic to them [80], especially when applied in synergy with biocides [81]. This could explain in part the observed Cu stress response observed in swarm center bacteria.

Both the gluconate permease and the glucose ABC transporter encoded by *gltKGF *[41] were up-regulated in swarm center. We speculate that swarm center cells are living in a glucose-depleted environment and that these systems are needed for a more efficient carbon acquisition. Here, it is important to note that while *pqq *(PA1985-89) and *gcd *(PA2290) genes were down-regulated in tip cells, they were "normally" expressed both in swarm center and non-swarming conditions. Thus, the oxidative pathway of glucose utilisation is not down-regulated in the swarm center, presumably because enough oxygen is available to cells in this condition.

### Tendril tip and swarm center bacteria constitute two distinct populations

Altogether, our results show that there is a global shutdown of transcripts associated with known virulence factors in swarming tendril tip bacteria (including *lecA*, *toxA*, *aprA*, *lasA*, *rhlAB*, *rhlC*, *pchFEDCBA*, *pvdH*, *pvdLG*). Swarm center and non-swarming control bacteria show a similar expression level for virulence-related genes (Table 3). Importantly, the up-regulating element common to all these factors is iron-restricted conditions, indicating that the iron-replete environment encountered by actively swarming cells explains, at least in part, their reduced expression of virulence determinants. At the same time, swarming tendril cells displayed high expression of genes associated with energy synthesis (*e.g. atpCDGAHFEBI*, and various cytochrome *c *oxidase subunits), and protein synthesis (ribomosal proteins, translation factors). Therefore, swarming cells at the tip of actively migrating tendrils are highly metabolically active cells that have reduced requirements for competition and nutrition acquisition factors.

A number of the down-regulated virulence factors in swarming tendril tip bacteria are also quorum sensing-controlled. Multicellular behaviour and cell-to-cell communication are often linked [3,82-84]. However, our results clearly show that nutritional factors, here especially iron, are key elements in the regulation of swarming motility; in contrast, we do not see a clear correlation with cell density. This agrees very well with the emerging concept that quorum sensing regulation cannot be separated from environmental factors, especially availability of nutrients [4,15,85].

H_2_O_2 _is generated during aerobic metabolism and is capable of damaging critical biomolecules. H_2_O_2 _production in bacteria usually mostly results from by-products of electron chain transport [86]. Our data however shows that only one gene belonging to that category, PA4133 encoding for a cytochrome *c *subunit, was up-regulated in swarm center. At the moment, it is not completely clear why oxidative stress response genes were up-regulated in swarm center and not in tendril tips where there is apparently a higher respiration activity. One possible explanation is the preferred glucose uptake and utilisation route taken by swarm tendril tip cells. Indeed, our data indicate that these cells preferentially use the phosphorylative instead of the oxidative pathway. Possibly, the latter generates more reactive oxygen species (ROS) that are dissimilated by superoxide dismutase (SOD) to generate H_2_O_2_. However, this explanation is only partial as it does not clarify why swarm center cells are different in this respect from cells of non-swarming colonies. We therefore advance another explanation integrating the observed oxidative and copper stress responses occurring in swarm center populations. H_2_O_2 _is a normal byproduct of oxidative metabolism and naturally reacts with reduced metal ions such Cu^2+^/Cu^+ ^to produce OH•/OOH• via the Haber-Weiss (aka Fenton-type) reaction [87,88], a strong oxidant well known to react with and damage biomolecules [89]. In our particular case, the input of Cu cations has to be coming from somewhere else than the impoverished medium on which swarm center bacteria lives. As reported in several studies, dead cells constitute an important component of a microbial biofilm [90-93]. More recently, Chang & Halverson (2009) reported a correlation between cell death and endogenous ROS accumulation in *P. putida *biofilms [94]. We propose that swarm center population comprises an important proportion of dead cells as is the case in *P. aeruginosa *biofilms (see additional file 6 for data supporting this hypothesis). These dead bacteria constitute an important reservoir of nutrient and metal species (such as Cu cations) diffusing into the live cells environment (reviewed in Harrison *et al*, (2007) [95]) and reacting with surrounding H_2_O_2 _to produce OH•/OOH• and therefore triggering an important oxidative stress. Catalase is also a heme containing redox enzyme and extreme iron limitation could prevent its normal function, thus possibly playing a role in the observed oxidative stress response.

There are important divergences between our transcriptomic data and the one presented by Overhage *et al*., (2008). This is likely explained mostly by the difference in our respective experimental designs, as they chose to compare their swarm tip bacteria against broth cultured bacteria. In our microarray experiment, swarm center and swarm tip bacteria were compared to a control colony grown on the same media solidified for a slightly longer drying period, thus avoiding gene expression differences specific to the surface vs. broth lifestyles. In consequence, both of our studies are not readily comparable. One possible explanation for their report of up-regulation of virulence factors in swarming colonies may be related to our observation that absence of restriction to growth results in diminished expression of virulence and colonization factors, such as extracellular proteases and siderophores. Differences in respective swarm plate media (M9DCAA vs. BM2) could also account for some of the observed differences.

**Figure 3:**
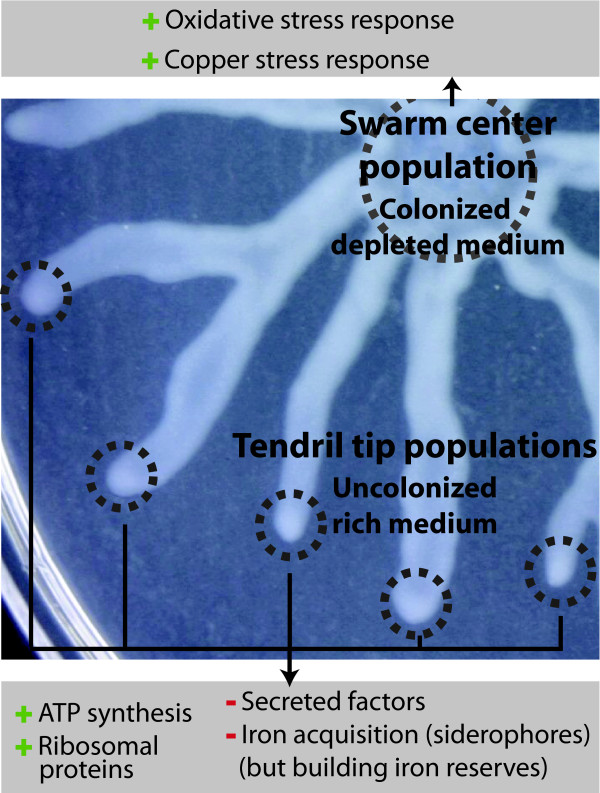
**Proposed model of the transcriptional dynamics displayed in tendril tips and swarm center of a *P. aeruginosa *swarming colony**. Tendril tip cells display an up-regulation of transcripts associated with energy production (ATP synthesis and cytochromes) and ribosomal proteins. At the same time, these cells down-regulate transcription associated with secreted factors (aka virulence factors) and iron acquisition. In contrast, swarm center cells live in a state in which oxidative and copper stress response transcripts are up-regulated.

In figure 3, we introduce a model in which we illustrate a swarming colony dynamics model in light of our data. We propose that tendril tip cells are specialized in colonization of pristine areas. Since these areas are free of other bacteria, subpopulations of bacteria whose task is to actually colonize do not need to express virulence/competition determinants. In such a model, their main task would be to rapidly spread from their inoculating point to appropriate immediate surrounding areas as fast as possible, leaving the duty of permanent colonization to swarm center bacteria who are expressing virulence factors and survival determinants. This model underscores the labour division and bacterial multicellularity of a swarming colony. Different subpopulations in the very same bacterial colony are an efficient way for a bacterial species to consolidate its control over an area.

## Conclusions

Besides the fact that *P. aeruginosa *absolutely needs a functional flagella, a low surface-tension medium and the production of rhamnolipids, very little is known about the regulatory features of swarming motility. In the present study, we report genes that are specifically expressed in swarm center or tendril tip populations of swarming colonies. We found that cells migrating at the tip of swarming tendrils are vigorously active as shown by the up-regulation of many genes involved in the electron respiratory chain transport and ATP production. In contrast, cells remaining in the center of the swarming colony express striking oxidative and copper stress responses. Compared to tendril tips, they also produce high amounts of transcripts of many secreted factors associated with virulence and iron acquisition. Iron and, more generally, nutrient acquisition genes were actually revealed to be a central aspect in our transcriptomic analysis.

We introduced a model in which labour division is an integral part of a swarming colony dynamics. This model reinforces the idea that swarming motility is essentially used by bacteria to colonize available nutrient-rich areas. We propose that *P. aeruginosa *swarming motility in itself is not a virulent behaviour, but rather an opportunity by a colony to rapidly spread and take control of a maximum of space thanks to swarm front's metabolically active (and fast moving) cells. Finally, our data suggest that the swarm center cells establish a more stable colony displaying a biofilm-like behaviour.

## Authors' contributions

JT designed and performed the transcript profiling and qRT-PCR experiments and carried out downstream data analysis. ED participated in the conception and supervised the design of the study, and performed data analysis. JT and ED wrote the manuscript. Both authors read and approved the final manuscript.

## Supplementary Material

Additional file 1 **Swarming motility video clip**. Movie clip of *Pseudomonas aeruginosa *PA14 swarming motility on M9DCAA medium solidified with 0.5% agar. The plate was incubated at 30°C. Video clip was constructed by assembling images taken every 3 minutes over a period of 21 hrs. and 45 min. with a Canon 10D camera equipped with a Canon Ultrasonic 28-80 mm lens. Click here for file

Additional file 2 **Differentially expressed genes of tendril tip, swarm center and non-swarming bacterial populations**. The complete list of genes differentially regulated in log_2 _fold-change with a *p*-value of 0.05 or lower. Click here for file

Additional file 3 **List of primers used in the qRT-PCR experiments**. Complete lists of primers used in the qRT-PCR experiments, including primer sequences are shown. Click here for file

Additional file 4 **Venn diagram representing differentially expressed genes for each conditions and possible combinations**. Genes in each categories (A to G) are listed in additional file #2 (excel file) under the "summary" tab. (↑) up-regulated genes; (↓) down-regulated genes. Group A: 58 genes up- and 134 down-regulated in tendril tip vs. non-swarming. Group B: 16 genes up- and 3 down-regulated in swarm center vs. non-swarming. Group C: 4 genes up- and 22 down-regulated in tendril tip vs. swarm center. Group D: 2 genes up- and 27 down-regulated in tendril tip vs. non-swarming and swarm center vs. non-swarming. Group E: 15 genes up- and 59 down-regulated in tendril tip vs. non-swarming and tendril tip vs. swarm center. Group F: *25 genes are down-regulated in tendril tip vs. swarm center while being up-regulated in swarm center vs. non-swarming control. Group G: 13 genes are down-regulated in tendril tip vs. non-swarming, tendril tip vs. swarm center and swarm center vs. non-swarming. Click here for file

Additional file 5 **Flagella observations**. Typical images of bacterial cells isolated from tendril tip, swarm center and non-swarming conditions. The flagella staining procedure was performed as described by Merritt and coworkers [96]. Click here for file

Additional file 6 **Swarm center population contains more dead cells than non-swarming bacteria**. Swarming and non-swarming colonies were grown as described in material and methods. For each conditions (swarm center and non-swarming), ten circular agar plugs of 0.75 cm diameter containing swarm center and non-swarming bacteria were extracted from plates and vigorously resuspended in 2 mL of sterile PBS buffer and serially diluted. For CFU count, 100 μL of each serial dilution was plated on TSB agar plates and incubated O/N at 37°C. To determine the dry weight, the remaining bacterial suspensions were placed in pre-weighed aluminum cups and incubated at 65°C for 4 hrs to allow water evaporation. Cups were weighed again to determine total dry weight. All experiments were performed in triplicates. Click here for file

## Aknowledgements

This study was supported by Natural Sciences and Engineering Research Council of Canada (NSERC) Discovery Grant No. 312478 to ED. JT was recipient of Ph.D. scholarships from the Fonds de la Recherche en Nature et Technologies (FQRNT) and from the Fondation Armand-Frappier. We thank David-Alexandre Fauvelle for useful comments.
